# An objective measure of response on whole-body MRI in metastatic hormone sensitive prostate cancer treated with androgen deprivation therapy, external beam radiotherapy, and radium-223

**DOI:** 10.1093/bjr/tqae005

**Published:** 2024-01-24

**Authors:** Valentina Giacometti, Arthur C Grey, Aaron J McCann, Kevin M Prise, Alan R Hounsell, Conor K McGarry, Philip G Turner, Joe M O’Sullivan

**Affiliations:** Advanced Radiotherapy Group, Patrick G. Johnston Centre for Cancer Research, Queen’s University Belfast, Belfast, Belfast, BT97 1NN, United Kingdom; Department of Imaging Services, Belfast Health & Social Care Trust, Belfast, BT9 7AB, United Kingdom; Department of Radiological Imaging & Protection Service, Regional Medical Physics Service, Belfast Health & Social Care Trust, Belfast, BT9 7AB, United Kingdom; Advanced Radiotherapy Group, Patrick G. Johnston Centre for Cancer Research, Queen’s University Belfast, Belfast, Belfast, BT97 1NN, United Kingdom; Advanced Radiotherapy Group, Patrick G. Johnston Centre for Cancer Research, Queen’s University Belfast, Belfast, Belfast, BT97 1NN, United Kingdom; Department of Radiotherapy Physics, Northern Ireland Cancer Centre, Belfast Health and Social Care Trust, Belfast, BT9 7AB, United Kingdom; Advanced Radiotherapy Group, Patrick G. Johnston Centre for Cancer Research, Queen’s University Belfast, Belfast, Belfast, BT97 1NN, United Kingdom; Department of Radiotherapy Physics, Northern Ireland Cancer Centre, Belfast Health and Social Care Trust, Belfast, BT9 7AB, United Kingdom; St Luke’s Cancer Centre, The Royal Hospital, Egerton Rd, Guildford GU2 7XX, United Kingdom; Advanced Radiotherapy Group, Patrick G. Johnston Centre for Cancer Research, Queen’s University Belfast, Belfast, Belfast, BT97 1NN, United Kingdom; Department of Clinical Oncology, Northern Ireland Cancer Centre, Belfast Health and Social Care Trust, Belfast, BT9 7AB, United Kingdom

**Keywords:** metastatic hormone sensitive prostate cancer, MRI intensity, biochemical progression, radiology response, vertebrae

## Abstract

**Objectives:**

The aim of this study was to generate an objective method to describe MRI data to assess response in the vertebrae of patients with metastatic hormone sensitive prostate cancer (mHSPC), treated with external beam radiation therapy and systemic therapy with Radium-223 and to correlate changes with clinical outcomes.

**Methods:**

Three sets of whole-body MRI (WBMRI) images were utilized from 25 patients from the neo-adjuvant Androgen Deprivation Therapy pelvic Radiotherapy and RADium-223 (ADRRAD) clinical trial: MRI1 (up to 28 days before Radium-223), MRI2, and MRI3 (2 and 6 months post completion of Radium-223). Radiological response was assessed based on post baseline MRI images. Vertebrae were semi-automatically contoured in the sagittal T1-weighted (T1w) acquisitions, MRI intensity was measured, and spinal cord was used to normalize the measurements. The relationship between MRI intensity vs time to biochemical progression and radiology response was investigated. Survival curves were generated and splitting measures for survival and biochemical progression investigated.

**Results:**

Using a splitting measure of 1.8, MRI1 was found to be a reliable quantitative indicator correlating with overall survival (*P* = 0.023) and biochemical progression (*P* = 0.014). MRI (3-1) and MRI (3-2) were found to be significant indicators for patients characterized by progressive/non-progressive disease (*P* = 0.021, *P* = 0.004) and biochemical progression within/after 12 months (*P* = 0.007, *P* = 0.001).

**Conclusions:**

We have identified a potentially useful objective measure of response on WBMRI of vertebrae containing bone metastases in mHSPC which correlates with survival/progression (prognostic) and radiology response (predictive).

**Advances in knowledge:**

Measurements of T1w WBMRI normalized intensity may allow identifying potentially useful response biomarkers correlating with survival, radiological response and biochemical progression.

## Introduction

Bone is the most common site of distant metastasis in advanced prostate cancer. Bone metastases have an incidence of ∼90% in metastatic castration resistant prostate cancer (mCRPC),^1–3^ and they are generated by the interaction between the tumor cells, and the bone microenvironment (osteoblasts, bone-forming cells; and osteoclasts, bone-resorbing cells). Bone metastases lead to fractures, increased pain, and increased mortality.^4^ Typically, when bone metastases are detected, the goal of therapy becomes to delay the disease progression, thus preserving or improving the patients’ quality of life and prolonging survival.^5^

The imaging techniques typically used to detect metastatic bone disease are isotope bone scan (IBS), magnetic resonance imaging (MRI), standard computer tomography (CT), single photon emission computed tomography, and positron emission tomography (PET) associated with CT and MRI. Sensitivity and specificity of these imaging modalities were reported in a review by Perez-Lopez et al^6^ and currently MRI is considered the most appropriate imaging modality to detect metastatic bone disease.^7^ MRI was shown to provide a superior capability than bone scintigraphy in detecting bone metastatic lesions,^8–10^ as confirmed in Barchetti et al^11^ where MRI sensitivity, specificity, and accuracy between 83%-99%, 82%-98%, and 84%-98%, respectively, depending on the MRI sequences acquired, were reported in patients with advanced prostate cancer. Prostate-specific membrane antigen (PSMA)-PET/CT is a valid alternative to MRI as a staging tool because it is considered the most sensitive technique for detecting low volume metastatic prostate cancer.^1^ However, advanced diseases castration-resistant prostate cancer are often treated with hormone therapy, and androgen suppression can alter the PSMA expression, thus limiting the capability of PSMA-PET/CT to correctly identify the metastasis and limiting the capability of this modality to assess response to therapy.^12^^,^^13^

Despite in “Response evaluation criteria in solid tumours” (RECIST, version 1.1) bone metastases were referred as a non-measurable disease,^14^ there has been an increasing interest in developing and validating a quantitative method to define the progression of bone metastasis after treatment.^1^^,^^15–19^ Tombal et al^15^ reported on the use of axial skeleton T1-weighted (T1w) and T2-weighted (T2w) MRI to evaluate tumor response in bone: bone lesions were measured (in mm) and the response was assessed on the basis of prostate-specific antigen (PSA) levels. Diffusion-weighted MRI (DWI) was also proven to be a valuable technique for monitoring the response of bone metastases because of its sensitivity to bone marrow cell density, bone marrow perfusion, and relative proportions of fat/water and marrow cells.^20^ Several studies have been published investigating the relationship between PSA level in response to treatment and changes in apparent diffusion coefficients (ADCs) in DWI.^16–18^^,^^21^ Reischauer et al^16^ reported on the first quantitative analysis correlating ADC values and patients’ response on a selected cohort of patients with advance prostate cancer, for which a decrease in PSA levels coincided with an increase in ADC. Perez-Lopez et al^17^ reported on the correlation between ADC and tumor diffusion volume (tDV) vs patient clinical response, assessed with Olaparib for patients with advanced prostate cancer. The assessment of bone metastases response often involves the measurements of metastasis volume,^22^^,^^23^ and in particular tDV was investigated for its promising results as prognostic value in prostate cancer.^24^

The aim of our study was to generate a new objective method to assess MRI response using the vertebrae of patients with metastatic hormone sensitive prostate cancer (mHSPC), by investigating the spine intensity measured on T1w MRI sequences. The correlation between T1w MRI intensity and clinical outcomes, including radiology MRI response, overall survival, and biochemical progression, was investigated with the goal of establishing novel prognostic, and predictive imaging biomarkers for mHSPC treated with radionuclide therapy.

## Materials and methods

### Study population and treatment

The study population consisted of 30 mHSPC patients from the neo-adjuvant Androgen Deprivation Therapy pelvic Radiotherapy and RADium-223 (Ra-223) clinical trial (ADRRAD). The trial protocol, patient details and clinical outcomes were described previously in.^25^ The prior article examined the combination of androgen deprivation therapy (ADT), upfront docetaxel, Ra-223, and external beam radiotherapy (EBRT) to prostate and pelvis and the results demonstrated that the treatment combination was well tolerated with encouraging efficacy results. In our study instead, we report the results of an exploratory quantitative analysis of the clinical images. We analyzed T1w MRI intensities to assess patient response on whole-body MRI (WBMRI) of vertebrae containing bone metastases in 25 of the 30 patients in the ADRRAD clinical trial cohort. Five patients were excluded from the study because one patient was deceased, one patient could not be imaged (claustrophobia), one patient had a screening MRI in a different hospital and the Sagittal T1 MRI in the initial WBMRI was missing for 2 patients.

These patients were diagnosed with prostate cancer stage T1-4 N0-1, M1b, they had at least 3 bone metastases visible on isotope bone scan, and life expectancy of at least 12 months. They were treated with ADT, 6 cycles of Docetaxel prior to EBRT (74 Gy/37 fractions to prostate and pelvic nodes), and 6 cycles of Ra-223 (55kBq/kg) at the Northern Ireland Cancer Centre (NICC) between 2016 and 2020. The first EBRT fraction and Ra-223 cycle were planned on the same day: the radiotherapy treatment was completed in 2 months, while the Ra-223 injections were once a month (6 months in total). Baseline bone scan and WBMRI were acquired to measure the diseases at baseline, WBMRI scans at 2 and 6 months post completion of Ra-223 were acquired to evaluate the tumor response. Patients deceased during the treatment (*n* = 2) and with WBMRI incomplete studies (*n* = 3) were unevaluable for analysis and excluded from this study.

### Whole-body MRI acquisition

Three sets of WBMRI were acquired: MRI1 baseline/screening scan performed at most 28 days before commencing EBRT and Radium-223, MRI2 performed 2 months post completion of Ra-223, and MRI3 performed 6 months post completion of Ra-223.

WBMRI were performed using a GE Optima MR450w 1.5 Tesla scanner (GE Medical System, Inc., Milwaukee, WI, United States, software release: SV25.0_R04/R05, receive coil: GEM posterior array) with a number of protocols, including T1w fast spin echo (TR = 618-657 ms, TE = 9.8-10.4 ms) and T2w fast recovery fast spin echo (TR = 3875-4173 ms, TE = 97.7-104.0 ms) sagittal plane imaging. The slice thickness was 3 mm, the field of view was 300 mm, and the acquisition type was 2-dimensional (2D). The main parameters characterizing T1w sequence are reported in Table 1.

**Table 1.** T1-weighted acquisition main parameters.

MRI acquisition type	2D
Magnetic field strength	1.5 T
Echo train length	3
Flip angle	160
Signal averages	1.5
Acquisition matrix	384/0/0/288
Pixel bandwidth	122.07

### Vertebrae segmentation and intensity measurements

**Figure 1.**
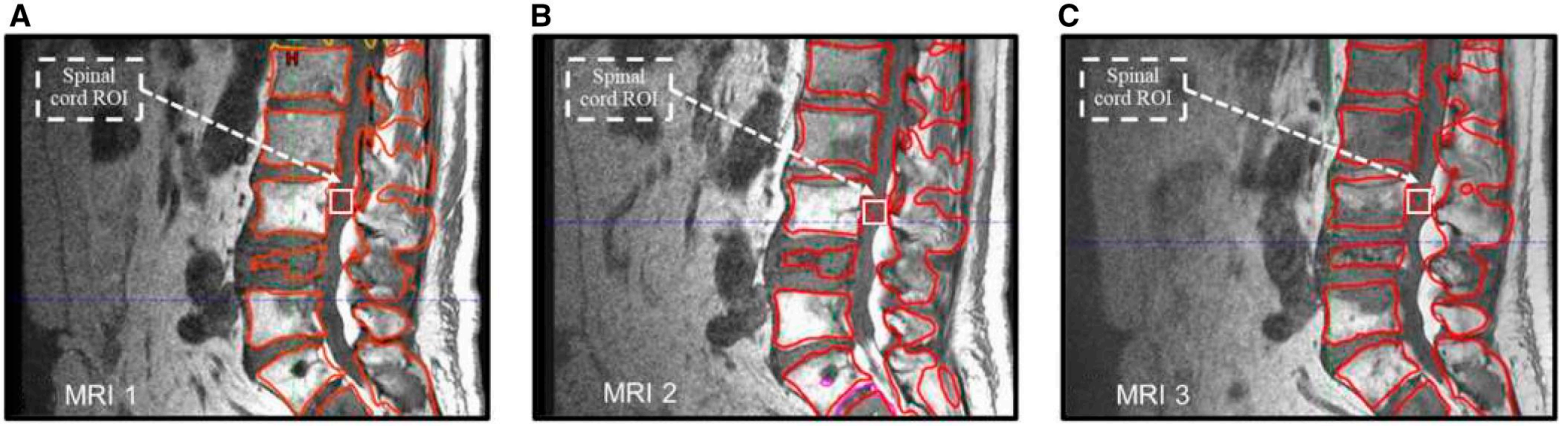
T1w MRI contours of the vertebrae outside the EBRT field for (A) MRI1, (B) MRI2, and (C) MRI3. The white square represents the spinal cord ROI of 1 × 1 cm^2^ chosen as reference for the normalization.

The WBMRI were imported into the Varian Eclipse (v13.5) (Varian Medical Systems, Palo Alto) treatment planning system and the vertebrae outside the EBRT field were semi-automatically contoured in the sagittal T1w sequences of the lumbar spine and the MRI intensity of the contoured vertebrae was measured. To compare the differences in intensity across MRI1, MRI2, and MRI3, the spinal cord intensity was chosen as a reference to normalize the measurements by selecting a region of interest of 1 × 1 cm^2^ located between the contoured vertebrae, as shown in Figure 1. This process is presented in detail in the Supplementary Material S1. The difference between normalized MRI intensities MRI (2-1), MRI (3-1), and MRI (3-2) were also calculated.

### Treatment response monitoring

The duration of survival and biochemical control were defined from the date of the first Ra-223 injection.

The survival and biochemical progression status were updated at a median follow-up of 36 months. Since this study focused on mHSPC patients’ response to treatment, biochemical progression and death by any cause were both included as progression events.

Biochemical progression was defined as 2 consecutive rises in PSA from nadir. The time to biochemical progression was used to group patients in early progression (within 12 months from the first Ra-223 injection) and late progression (at least 12 months after the first Ra-223 injection). Patients were also analyzed for biochemical progression within/after 24 months from the first Ra-223 injection.

Radiology MRI response was assessed based on post baseline MRI images. Patients showing partial, complete, or stable responses were classified as “non-progressive” disease, while non-responding patients were classified as “progressive” disease.

### Statistical analysis

Statistically significant differences between normalized MRI intensities for patients grouped into progressive/non-progressive disease and early/late biochemical progression were investigated by performing Mann-Whitney and Student’s *t*-test (results presented in Supplementary Material S2). The normality of the data samples was tested with the Shapiro-Wilk test. If normally distributed, the *F*-test was performed to test the equality of variance across groups.

Kaplan-Meier analysis was used to investigate survival and biochemical progression probability, and splitting measures for the normalized MRI intensities were evaluated. The comparison between the discriminative ability of MRI1, 2, and 3 in predicting survival and biochemical progression was performed by determining the area under the receiver operating characteristic curve (ROC), knowing the overall survival and biochemical progression status. The method established by DeLong et al^26^ was used to compare the ROC curves for MRI 1, 2, and 3 and determining if their difference was statistically significant.

Statistical analysis was performed using a statistics software package (R, http://www.R-project.org/); all tests were 2-sided, with significance set at *P*-value <0.05.

## Results

In the present study, T1w MRI intensity of the spine between T11 and S2 vertebrae was measured in 25 mHSPC patients (mean age, 65 years; range 48-82 years). Nine (36%) and 16 (64%) patients in the cohort had ≤10 and >20 bone metastases visible on isotope bone scan at baseline, respectively. The characteristics of the study population at baseline are summarized in Table 2.

**Table 2.** Baseline characteristic of the study population (*n* = 25).

Parameter at baseline	Median	Range
Age	64.4	48.0-82.0
Prostate-specific antigen (PSA) (ng/mL)	0.5	0.0-6.3
Alkaline phosphatase (ALP) (U/L)	88.0	53.0-171.0
Lactate dehydrogenase (LDH) (U/L)	205.5	96.0-349.0

Eleven (44%) of the 25 patients were alive at the time of the data analysis, 14 (56%) died during the follow-up period. The median overall survival was 51 months and the median time to biochemical progression was 23 months. The number of biochemically progressed patients was 6 (24%) within 12 months, 7 (28%) between 12 and 24 months, 12 (48%) after 24 months. PSA relapse was recorded for 17 (68%) of the 25 patients, 6 within 12 months, 4 between 12 and 24 months, 7 after 24 months. The assessment based on the MRI response counted 10 (40%) patients with progressive diseases and 15 (60%) with non-progressive disease (6 partial response, 7 complete response, and 2 stable disease).

**Figure 2.**
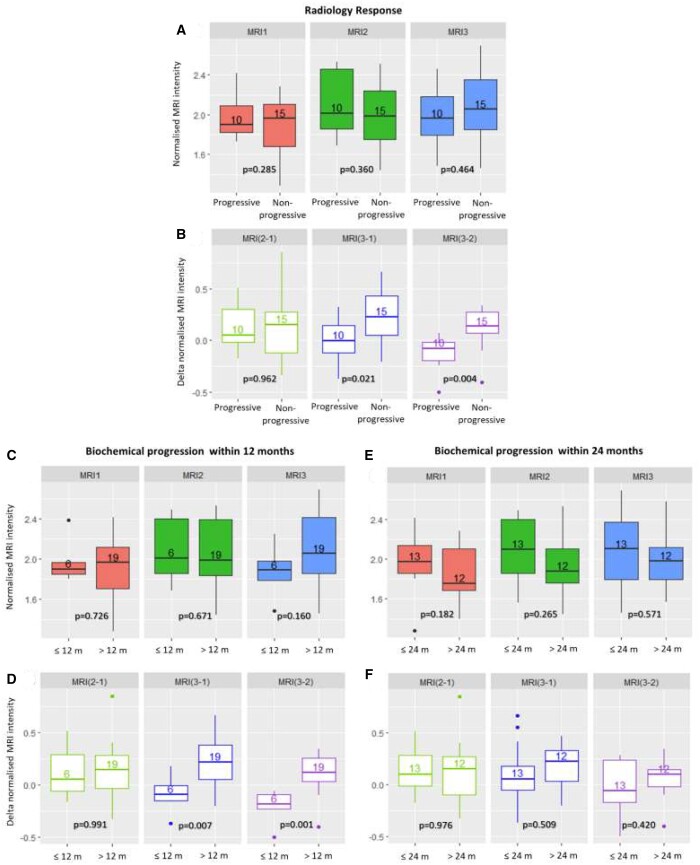
MRI1, 2, and 3 and MRI (2-1), MRI (3-1), and MRI (3-2) normalized intensities grouped according to (A, B) radiology response (progressive: *n* = 10; non-progressive: *n* = 15), (C, D) biochemical progression within 12 months (≤12 months: *n* = 6; >12 months: *n* = 19), (E, F) biochemical progression within 24 months (≤24 months: *n* = 13; >24 months: *n* = 12). The corresponding *P*-values are reported underneath. The number of patients in each group is written inside the boxplot. The upper and lower whiskers are plotted by extending the whiskers to the furthest data point within 1.5 times the interquartile range from each box end. Abbreviation: m = months.

Figure 2 shows MRI1, 2, 3 and MRI (2-1), MRI (3-1), and MRI (3-2) normalized intensities grouped according to the radiology MRI response (Figure 2A and B) and time to biochemical progression (12 months: Figure 2C and D—24 months: Figure 2E and F). These boxplots aimed to show if MRI normalized intensity could be associated with a group of responding or non-responding patients. To quantify the differences among the boxplots presented, *P*-values were calculated (*P* < 0.05 means that the data presented in the boxplots are statistically significantly different).

MRI1, 2, 3 normalized intensities were not significantly different among the 3 groups. However, the variation between normalized intensities MRI (3-1) and MRI (3-2) were statistically significantly different for progressive vs non-progressive (*P* = 0.021 and *P* = 0.004) and for time to biochemical progression ≤12 months vs >12 months (*P* = 0.007 and *P* = 0.001). No statistically significant differences were observed for time to biochemical progression ≤24 vs >24 months. A detailed summary of the statistics of these data is presented in the Supplementary Material S2.

**Figure 3.**
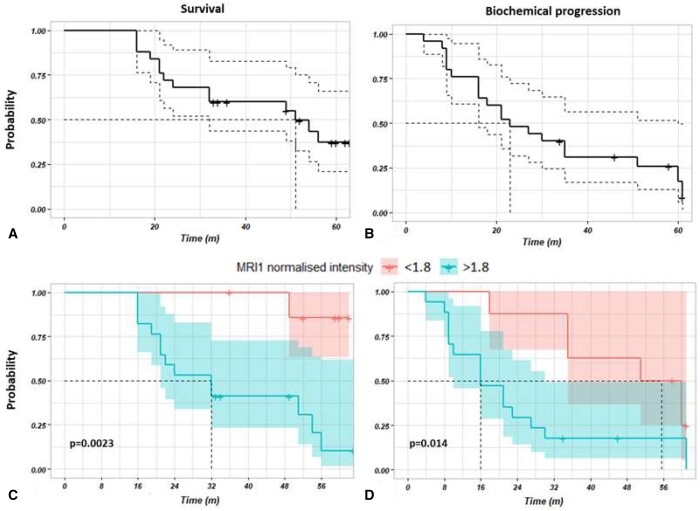
Kaplan-Meier survival curves of (A) survival and (B) biochemical progression probability vs time to censoring/death and time to censoring/progression, respectively; (C) survival and (D) biochemical progression probability split according to the optimal splitting value of 1.8 for MRI1. The dash black line indicates the median survival/progression.

Figure 3 shows the Kaplan-Meier survival curves generated for the 25 patients: survival (Figure 3A) and biochemical progression (Figure 3B) probability were plotted vs time to censoring/death and time to censoring/progression, respectively.

To assess the prognostic performance of MRI imaging, splitting measures for the normalized MRI intensities were investigated to significantly separate the probability curves. In order to determine the optimal splitting values for MRI 1, 2, and 3, 2 criteria had to be fulfilled: (1) *P*-value <0.05, (2) balance among the number of patients in the 2 separated groups, determined by accepting the separation only if the ratio between the number of patients per group was <3. An in-depth description of the MRI splitting values investigation is presented in the Supplementary Material S3.

The best splitting value for survival probability were 1.8 (*P* = 0.002), 2.02 (*P* = 0.053), 2.10 (*P* = 0.259) for MRI1, 2 and 3, respectively, and for biochemical progression probability were 1.8 (0.014), 2.02 (*P* = 0.340), 2.00 (*P* = 0.204) for MRI1, 2, and 3, respectively. The reported results are statistically significant (*P* < 0.05) both for survival and progression probability only for MRI1, but not for MRI2 and MRI3. Therefore, an optimal splitting value of 1.8 was chosen for MRI1, and the split in survival and biochemical progression probability curves was generated (Figure 3C and D).

**Figure 4.**
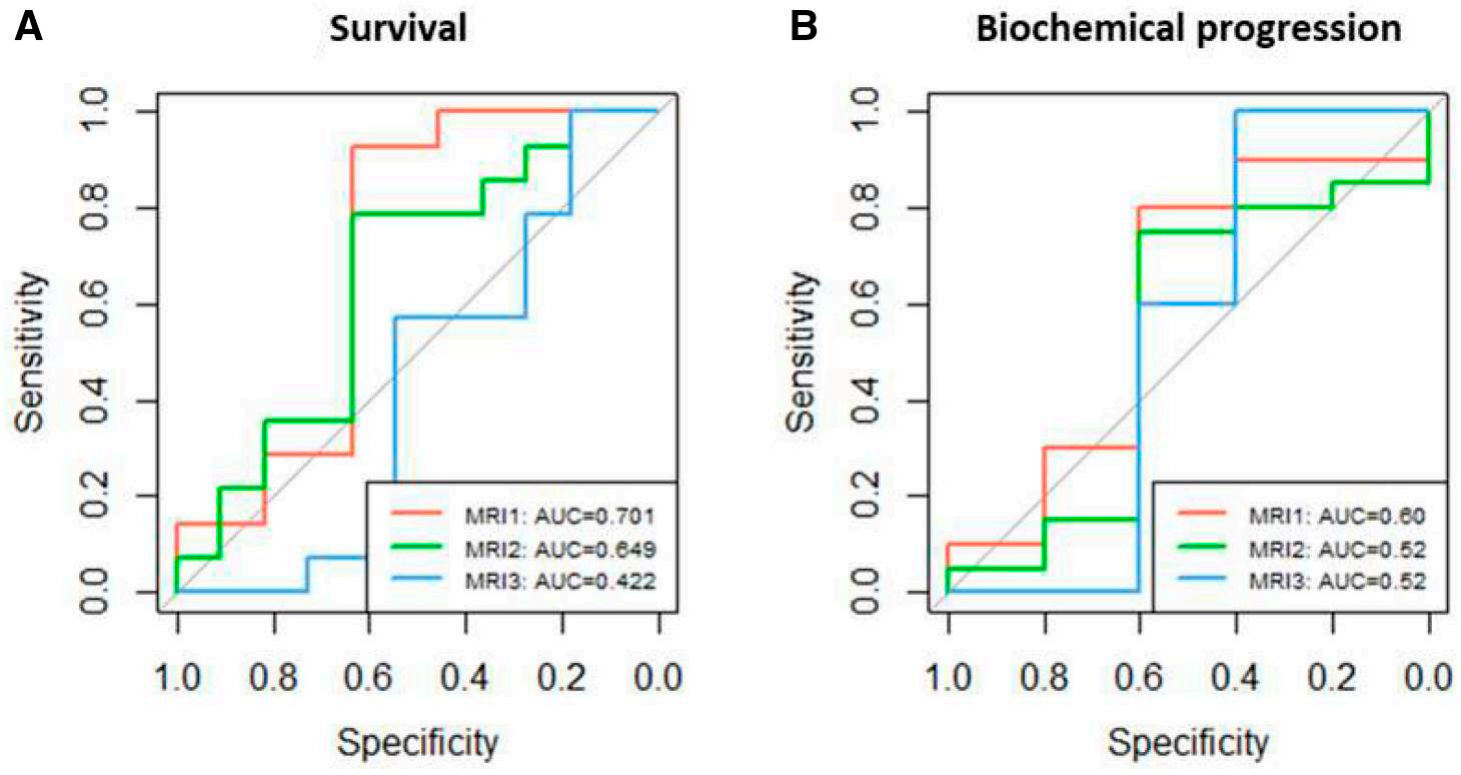
ROC curves for (A) survival and (B) biochemical progression of MRI1, MRI2, and MRI3. The area under the curve (AUC) for MRI1 is consistently slightly higher than MRI2 and MRI3. For survival, ROC: 0.701 vs 0.649, *P* = 0.756; 0.649 vs 0.422, *P* = 0.325; 0.701 vs 0.422, *P* = 0.152. For biochemical progression, ROC: 0.60 vs 0.52, *P* = 0.717; 0.52 vs 0.52, *P* = 1.000; 0.60 vs 0.52, *P* = 0.817.

These results are confirmed by the ROC analysis of survival and progression vs MRI1, 2, and 3, presented in Figure 4. The area under the curve for MRI1 is consistently slightly higher than MRI2 and 3 but these differences are not statistically significant (*P* > 0.05).

## Discussion

More than 90% of patients with advanced prostate cancer develop bone metastases^16^ particularly in the spine and pelvis region. Several imaging techniques are currently used to identify bone metastases, and particularly the use of MRI as non-invasive technique for assessing the treatment response has been widely investigated.^1^^,^^5^^,^^15–17^^,^^19^^,^^21^^,^^25^^,^^27^ In this study, we present an objective measure of response based on WBMRI of vertebrae containing bone metastases in mHSPC which correlates with overall survival and radiological response. An optimal splitting value of.1.8 for survival and biochemical progression probability was determined for the baseline MRI. MRI response assessed by a radiologist and patients’ biochemical progression were found to be significantly different according to the variation of normalized MRI intensities during the treatment.

Bone scan and bone scan index have been widely investigated as a prognostic factor in patients with prostate cancer^26,28^–30 and a comparison between MRI and IBS was reported in.^24^ Bone scanning is the recommended imaging technique by the Prostate Cancer Working Group 3 (PCWG3)^30^ for assessing response in skeletal disease using the 2 + 2 method. Although, the RECIST criteria^14^ is not ideal to identify metastatic bone diseases in prostate cancer patients because it classifies the osteoblastic bone metastases as non-measurable, and these are the most encountered metastases in prostate cancer. Consequently, standard bone scans are inadequate for measuring bone lesions and existing technology limitations require RECIST to classify bone metastases as non-measurable disease. The PCWG3 also underlined that bone scan and PSA changes do not always accurately represent objective changes in tumor burden, highlighting the difficulty in identifying if the detection of new sites of disease on a follow-up bone scan represents flare of a pre-existing subclinical metastatic lesions or a true transition to a metastatic state.^30^^,^^31^ Therefore, different imaging modalities could provide better information to assess bone metastases behavior.

The first attempt in investigating bone metastases response to treatment by using MRI was conducted by Tombal et al^15^ who classified spinal and pelvi-femoral bone metastases according to their bone marrow involvement by visually checking T1w and T2w MRIs. The authors identified 3 MRI patterns (normal, focal metastatic lesion, diffuse marrow infiltration) and measured the size of the lesions at baseline and 6 months after chemotherapy to investigate the correlation between MRI patient response, PSA levels and changes in soft tissue lesions. Similar to our study, the authors concluded that MRI is an objective tool that can be used to quantitatively measure metastatic disease, and MRI and PSA evaluation of response agreed for 14 out of 20 patients. Padhani et al^5^ also described 4 patterns of change in response to therapy for bone marrow lesions and associated them with ADC changes respect to cutoff values previously determined. The authors recommended establishment of a cutoff value by examining untreated patients with the same imaging protocol and underlined that these values most likely would be dependent on the type of cancer. Our findings support these results, as we chose to normalize each MRI spine intensity on a patient specific base and not using one single normalization factor to avoid bias since the acquired images are sensitive variations in the MRI acquisition (different length acquired, imaging artifacts, etc.).

However, there is a paucity of data quantitatively investigating classification of different patient responses for prostate cancer bone metastases and at present, ADC is the only parameter used to numerically quantify MRI changes in metastatic bone disease. Cappabianca et al^25^ identified 4 patterns response associated to ADC and *b*-value in DW MRI corresponding to 3 different responses to treatment (disease progression, indeterminate and good response). A study by Reischauer et al^16^ focused on developing and validating a non-invasive response biomarker for prostate cancer bone metastases based on DWI assessment of response. A strong correlation between ADC levels and PSA variation highlighted the potential of quantitative ADC map for assessing treatment response. Similarly, Perez-Lopez et al^17^ showed that bone metastases response could be assessed with WB DWI by measuring the changes in metastases volume and correspondent median ADC values after 12 weeks of treatment (patients treated with polymerase inhibitor Olaparib). In our study we reported on the use of a new parameter (T1w MRI normalized intensity) to numerically quantify MRI changes. T1w MRI normalized intensity was used to assess patients’ response, and accordingly to what was previously published in literature, correlations with overall survival and biochemical progression were investigated, thus showing how T1w MRI could potentially be used as a predictive and prognostic biomarker in studies where DWI is not available. On the other hand, if both T1w MRI and DWI are available, it could be possible to further investigate what T1w MRI normalized intensity changes are indicating in terms of bone structure (bone marrow changes, metastases changes, etc.).

In contrast to other studies,^14^^,^^17^ the measurements of the size of bone metastases was not required to assess the patient response in our work, thus reducing contouring time and avoiding contouring bias. The introduction of a semi-automatic thresholding technique for outlining the spine and the measurement of T1w MRI intensity normalized with spinal cord intensity improves the objectivity, allows repeatability, and provides a systematic and faster way to perform these measurements (Supplementary Material S1).

The difference in normalized intensity between different time-points in the treatment revealed a significant correlation with progressive/non-progressive diseases and with early/late progression (*P* < 0.005) thus suggesting that T1w MRI normalized intensity could be a potential tool to describe the patient response. The single normalized response MRI1, MRI2, and MRI3 provided novel and interesting results in terms of survival and biochemical progression probability and an optimal T1w MRI splitting value of 1.8 for separating survival and biochemical progression probability was established (Supplementary Material S3). These preliminary results are very promising because they suggest the possibility of predicting how well the bone metastasis would respond to the treatment in terms of survival or progression from the baseline MRI. In fact, it was not foreseen to find a significant splitting value; nevertheless, a value was observed for intensity values of MRI1. Therefore, it is necessary to validate how to establish the splitting value on a bigger cohort, so that the patients’ treatment could be personalized according to the predicted response.

Potential limitations of the study must be also acknowledged. First, the dataset here presented is relatively small (*n* = 25), and a larger dataset is needed to validate the presented findings. Moreover, this dataset is unique, with 3 WBMRI acquired during the whole treatment, therefore finding similar dataset may be challenging.^3^ Second, the numbers of vertebrae imaged in the acquired sagittal MRI sequences were not consistent. Therefore, some of the contoured vertebrae were excluded from the measurements to ensure that the same number of vertebrae were compared for each individual patient when measuring the MRI intensity. Third, some patients experienced vertebrae compressions and fractures during the treatment causing the semi-automatic contours decreasing its accuracy, and consequently manual corrections were required to correct suboptimal contours. Finally, the lack of a biological interpretation of T1w MRI intensity is a challenge for establishing this quantity as an imaging biomarker.^32^

Despite these limitations, our study presents the first quantitative investigation assessing the bone metastases response based only on T1w MRI intensity for patients with mHSPC and focusing on the potential of the measurements as a predictive factor.

In this study, a splitting value was retrospectively determined on the baseline T1w MRI intensity, based on patients’ overall survival and biochemical progression. This approach should be validated by using an external dataset, in order to determine if this value could be used as a prognostic biomarker. In fact, T1w MRI intensity is specific to the scanner and sequence used, and it does not represent an intrinsic property of the tissue whereby the accuracy and utility of the measurement could be fully characterized only with reference to some ground truth (similarly to what has been done, for example for the liver iron content calculated from R2 at a given field strength^33^). Moreover, an investigation on the optimal time-points when acquiring follow-up MRIs is needed in order to fully exploit the potential of the difference between normalized MRI intensities as a predictive biomarker. This analysis should be extended also to the vertebrae inside the EBRT field, in order to study the impact of the dose on the bone metastases response to the treatment.

Finally, investigating a potential correlation between normalized MRI intensity in T1w WBMRI and ADCs in WB DWI, in terms of overall survival and biochemical progression should be considered for further studies. A range of quantitative MRI (qMRI) biomarkers such as quantitative T1 mapping via relaxometry are currently making the transition from research into clinical use.^34^^,^^35^ Future studies incorporating the rigorous standards required for qMRI, and the use of a reference test object such as those developed by NIST,^36^ could potentially improve the accuracy and precision of clinical trial outcomes, and thus provide a better understanding of T1 as prognostic and predictive imaging biomarker.

## Conclusion

Patients with advanced prostate cancer often develop bone metastases, leading to bone pain, skeletal fracture, and increased mortality.

In this study, a quantitative analysis of the T1w MRI intensity used to assess the patient response to treatment has been presented. The method identified a potentially valuable objective measure of response on WBMRI of vertebrae containing bone metastases in mHSPC which correlates with radiological response, patients’ biochemical progression, time to survival, and time to progression.

Further studies are needed to validate these findings in a larger dataset.

## Supplementary Material

tqae005_Supplementary_Data
