# Supplementary material for: An objective measure of response on whole-body MRI in metastatic hormone sensitive prostate cancer treated with androgen deprivation therapy, external beam radiotherapy, and radium-223
Source: Br J Radiol. 2024 Jan 24;97(1156):794–802. doi: 10.1093/bjr/tqae005 (PMC11027342; doi:10.1093/bjr/tqae005)
Supplement: tqae005_Supplementary_Data [file tqae005_supplementary_data.docx]

**S1. Spine contouring and T1w MRI intensity measurement**

The spine was semi-automatically contoured in sagittal T1w and T2w MRI sequences of the lumbar spine by implementing the following steps in the Varian Eclipse (v13.5) (Varian Medical Systems, Palo Alto) treatment planning system. The main steps of this process are shown in Figure S1.

1. Rigid registration of planning CT and sagittal T1w and T2w MRI.
2. Threshold applied to the sagittal T2w MRI in order to delineate the contours between spine and disks, as shown in Figure S1(a).
3. Threshold applied to the planning CT in order to delineate the contours between spine and back muscles/abdomen, as shown in Figure S1(b).
4. Intersection between the contours delineated at step 2 and 3, and post processing to delete gaps, as shown in Figure S1(c).
5. Visual inspection and (if necessary) manual correction of the automatic contours generated on the sagittal T2w MRI.
6. Copy of the inspected spine contours onto the sagittal T1w MRI, as shown in Figure S1(d).
7. Spine contours reviewed by an oncologist.
8. Cropping the contours of the spine to include only the vertebrae outside the external beam radiotherapy (EBRT).
9. Average MRI intensity measured for the spine contoured in the T1w MRI.
10. Spinal cord region of interest (ROI) of 1×1 cm^2^ selected in the T1w MRI to normalise the spine MRI intensity measured at step 9, as shown in Figure S1(d). Spinal cord ROI was chosen for its proximity to the contoured vertebrae to mitigate the effects of hardware related signal variation.
11. Normalised intensity signal calculated by dividing the average T1w signal measured at step 9 by the average intensity value within the spinal cord ROI.

**
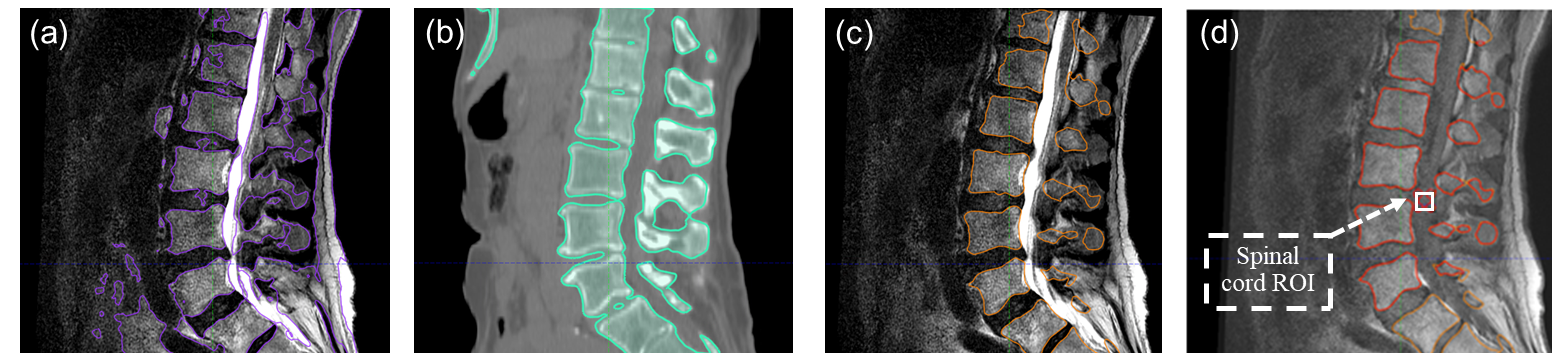
**

Figure S1: Schematic summary of steps 1-10 to semi-automatically contour the spine and to measure its MRI intensity.

(a) Threshold applied to the sagittal T2w MRI.

(b) Threshold applied to the planning CT.

(c) Intersection between T2w MRI and planning CT contours.

(d) T1w MRI contours of all the vertebrae (orange) and of the vertebrae outside the EBRT field (red). The 1×1cm^2^ spinal cord ROI chosen as reference for the normalisation is indicated with an arrow and a white square.

**S2. Patient response summary statistics**

A detailed summary of the statistics of MIR1, 2, 3, MRI(2-1), MRI(3-1) and MRI(3-2) grouped according to radiology MRI response (progressive/non-progressive), biochemical progression (within/after 12 and 24 months) and status (alive and biochemical progression yes/no) are displayed in Table S2.1, S2.2 and S2.3, respectively. Statistically significant results are achieved when p-value<0.05.

Table S2.1: Summary statistics for normalised MRI1, 2 and 3 and MRI(2-1), MRI(3-1) and MRI(3-2) intensities vs progressive/non-progressive disease. The p-values are calculated with Mann-Whitney or Student’s t-test. Abbreviations: m=months, n= number, SD = standard deviation, min= minimum, max= maximum. (*) statistically significant results.

|  | **Radiology response** | **n** | **mean** | **SD** | **median** | **min** | **max** | **p-value** |
| --- | --- | --- | --- | --- | --- | --- | --- | --- |
| **MRI1** | ***Progressive*** | 10 | 1.990 | 0.243 | 1.900 | 1.730 | 2.410 | 0.285 |
|  | ***Non-progressive*** | 15 | 1.870 | 0.298 | 1.960 | 1.280 | 2.280 |  |
| **MRI2** | ***Progressive*** | 10 | 2.110 | 0.333 | 2.010 | 1.690 | 2.530 | 0.360 |
|  | ***Non-progressive*** | 15 | 1.980 | 0.344 | 1.990 | 1.440 | 2.510 |  |
| **MRI3** | ***Progressive*** | 10 | 1.990 | 0.309 | 1.960 | 1.480 | 2.460 | 0.464 |
|  | ***Non-progressive*** | 15 | 2.100 | 0.381 | 2.050 | 1.460 | 2.690 |  |
| **MRI(2-1)** | ***Progressive*** | 10 | 0.123 | 0.226 | 0.052 | -0.170 | 0.508 | 0.962 |
|  | ***Non-progressive*** | 15 | 0.117 | 0.314 | 0.153 | -0.333 | 0.848 |  |
| **MRI(3-1)** | ***Progressive*** | 10 | 0.003 | 0.202 | -0.002 | -0.370 | 0.320 | 0.021* |
|  | ***Non-progressive*** | 15 | 0.235 | 0.247 | 0.228 | -0.210 | 0.661 |  |
| **MRI(3-2)** | ***Progressive*** | 10 | -0.120 | 0.170 | -0.076 | -0.501 | 0.073 | 0.004* |
|  | ***Non-progressive*** | 15 | 0.118 | 0.197 | 0.136 | -0.404 | 0.337 |  |

Table S2.2: Summary statistics for normalised MRI1, 2 and 3 and MRI(2-1), MRI(3-1) and MRI(3-2) intensities vs biochemical progression within 12 and 24 months. The p-values are calculated with Mann-Whitney or Student’s t-test. Abbreviations: m=months, n= number, SD = standard deviation, min= minimum, max= maximum. (*) statistically significant results.

|  | **Biochemical progression** | **n** | **mean** | **SD** | **median** | **min** | **max** | **p-value** | **Biochemical progression** | **n** | **mean** | **SD** | **median** | **min** | **max** | **p-value** |
| --- | --- | --- | --- | --- | --- | --- | --- | --- | --- | --- | --- | --- | --- | --- | --- | --- |
| **MRI1** | ***≤ 12 m*** | 6 | 1.970 | 0.214 | 1.900 | 1.800 | 2.390 | 0.726 | ***≤ 24 m*** | 13 | 1.990 | 0.285 | 1.970 | 1.280 | 2.410 | 0.182 |
|  | ***> 12 m*** | 19 | 1.900 | 0.299 | 1.960 | 1.280 | 2.410 |  | ***> 24 m*** | 12 | 1.840 | 0.261 | 1.750 | 1.400 | 2.280 |  |
| **MRI2** | ***≤ 12 m*** | 6 | 2.090 | 0.342 | 2.010 | 1.690 | 2.490 | 0.671 | ***≤24 m*** | 13 | 1.950 | 0.363 | 1.880 | 1.440 | 2.530 | 0.265 |
|  | ***> 12 m*** | 19 | 2.020 | 0.346 | 1.990 | 1.440 | 2.530 |  | ***> 24 m*** | 12 | 2.110 | 0.311 | 2.100 | 1.560 | 2.490 |  |
| **MRI3** | ***≤ 12 m*** | 6 | 1.880 | 0.255 | 1.890 | 1.480 | 2.250 | 0.160 | ***≤ 24 m*** | 13 | 2.100 | 0.399 | 2.100 | 1.460 | 2.690 | 0.571 |
|  | ***> 12 m*** | 19 | 2.110 | 0.364 | 2.050 | 1.460 | 2.690 |  | ***> 24 m*** | 12 | 2.010 | 0.303 | 1.980 | 1.570 | 2.580 |  |
| **MRI(2-1)** | ***≤ 12 m*** | 6 | 0.118 | 0.263 | 0.052 | -0.170 | 0.508 | 0.991 | ***≤ 24 m*** | 13 | 0.121 | 0.223 | 0.099 | -0.183 | 0.508 | 0.976 |
|  | ***> 12 m*** | 19 | 0.120 | 0.289 | 0.143 | -0.333 | 0.848 |  | ***> 24 m*** | 12 | 0.118 | 0.337 | 0.148 | -0.333 | 0.848 |  |
| **MRI(3-1)** | ***≤ 12 m*** | 6 | -0.090 | 0.183 | -0.098 | -0.370 | 0.178 | 0.007* | ***≤ 24 m*** | 13 | 0.109 | 0.291 | 0.053 | -0.370 | 0.661 | 0.509 |
|  | ***> 12 m*** | 19 | 0.215 | 0.230 | 0.215 | -0.210 | 0.661 |  | ***> 24 m*** | 12 | 0.178 | 0.213 | 0.222 | -0.210 | 0.466 |  |
| **MRI(3-2)** | ***≤ 12 m*** | 6 | -0.208 | 0.161 | -0.188 | -0.501 | -0.060 | 0.001* | ***≤ 24 m*** | 13 | -0.012 | 0.240 | -0.060 | -0.501 | 0.279 | 0.420 |
|  | ***> 12 m*** | 19 | 0.096 | 0.181 | 0.115 | -0.404 | 0.337 |  | ***> 24 m*** | 12 | 0.060 | 0.195 | 0.094 | -0.404 | 0.337 |  |

Table S2.3: Summary statistics for normalised MRI1, 2 and 3 and MRI(2-1), MRI(3-1) and MRI(3-2) intensities vs survived and biochemically progressed patients. The p-values are calculated with Mann-Whitney or Student’s t-test. Abbreviations: m=months, n= number, SD = standard deviation, min= minimum, max= maximum, y= yes, n=no.

|  | **Alive** | **n** | **mean** | **SD** | **median** | **min** | **max** | **p-value** | **Biochemical progression** | **n** | **mean** | **SD** | **median** | **min** | **max** | **p-value** |
| --- | --- | --- | --- | --- | --- | --- | --- | --- | --- | --- | --- | --- | --- | --- | --- | --- |
| **MRI1** | ***Y*** | 11 | 2.000 | 0.327 | 1.730 | 1.280 | 2.280 | 0.075 | ***Y*** | 20 | 1.920 | 0.282 | 1.950 | 1.280 | 2.410 | 0.767 |
|  | ***N*** | 14 | 1.800 | 0.206 | 2.000 | 1.730 | 2.410 |  | ***N*** | 5 | 1.880 | 0.296 | 1.730 | 1.610 | 2.280 |  |
| **MRI2** | ***Y*** | 11 | 1.890 | 0.347 | 1.890 | 1.440 | 2.510 | 0.060 | ***Y*** | 20 | 2.040 | 0.364 | 2.020 | 1.440 | 2.530 | 0.919 |
|  | ***N*** | 14 | 2.150 | 0.298 | 2.130 | 1.690 | 2.530 |  | ***N*** | 5 | 2.020 | 0.247 | 1.900 | 1.880 | 2.460 |  |
| **MRI3** | ***Y*** | 11 | 1.970 | 0.333 | 2.020 | 1.460 | 2.580 | 0.285 | ***Y*** | 20 | 2.060 | 0.385 | 1.980 | 1.460 | 2.690 | 0.931 |
|  | ***N*** | 14 | 2.130 | 0.362 | 2.040 | 1.480 | 2.690 |  | ***N*** | 5 | 2.040 | 0.191 | 2.040 | 1.780 | 2.320 |  |
| **MRI(2-1)** | ***Y*** | 11 | 0.088 | 0.355 | 0.153 | -0.333 | 0.848 | 0.628 | ***Y*** | 20 | 0.114 | 0.230 | 0.115 | -0.333 | 0.508 | 0.906 |
|  | ***N*** | 14 | 0.144 | 0.209 | 0.115 | -0.170 | 0.508 |  | ***N*** | 5 | 0.140 | 0.457 | 0.153 | -0.297 | 0.848 |  |
| **MRI(3-1)** | ***Y*** | 11 | 0.167 | 0.213 | 0.177 | -0.210 | 0.466 | 0.671 | ***Y*** | 20 | 0.137 | 0.266 | 0.117 | -0.370 | 0.661 | 0.836 |
|  | ***N*** | 14 | 0.122 | 0.287 | 0.050 | -0.370 | 0.661 |  | ***N*** | 5 | 0.164 | 0.217 | 0.052 | -0.059 | 0.444 |  |
| **MRI(3-2)** | ***Y*** | 11 | 0.079 | 0.211 | 0.124 | -0.404 | 0.337 | 0.261 | ***Y*** | 20 | 0.023 | 0.207 | 0.065 | -0.501 | 0.287 | 0.995 |
|  | ***N*** | 14 | -0.022 | 0.220 | -0.031 | -0.501 | 0.279 |  | ***N*** | 5 | 0.023 | 0.285 | 0.115 | -0.404 | 0.337 |  |

**S3. Optimal splitting values for MRI 1, 2, and 3.**

The splitting values for separating survival and biochemical progression probability curves for MRI1, MRI2 and MRI3 were investigated.

First, mean and standard deviation of all patients normalised responses for MRI1, MR2 and MR3 were calculated, and the results were 1.9±0.3, 2.0±0.4, and 2.1±0.4, respectively. The splitting value had to be found among the normalised responses, therefore a range of values was established: 1.6 was the lowest value (lowest mean -1 standard deviation), and 2.5 was the highest value (highest mean +1 standard deviation). To investigate the significance of the splitting values, the p-values for each value within this range, with steps of 0.02, were calculated. As shown in figure S3, significant p-values (< 0.05) were found only for MRI1. Both the survival and the progression probability split are statistically significant for MRI1 with a splitting value of 1.8.


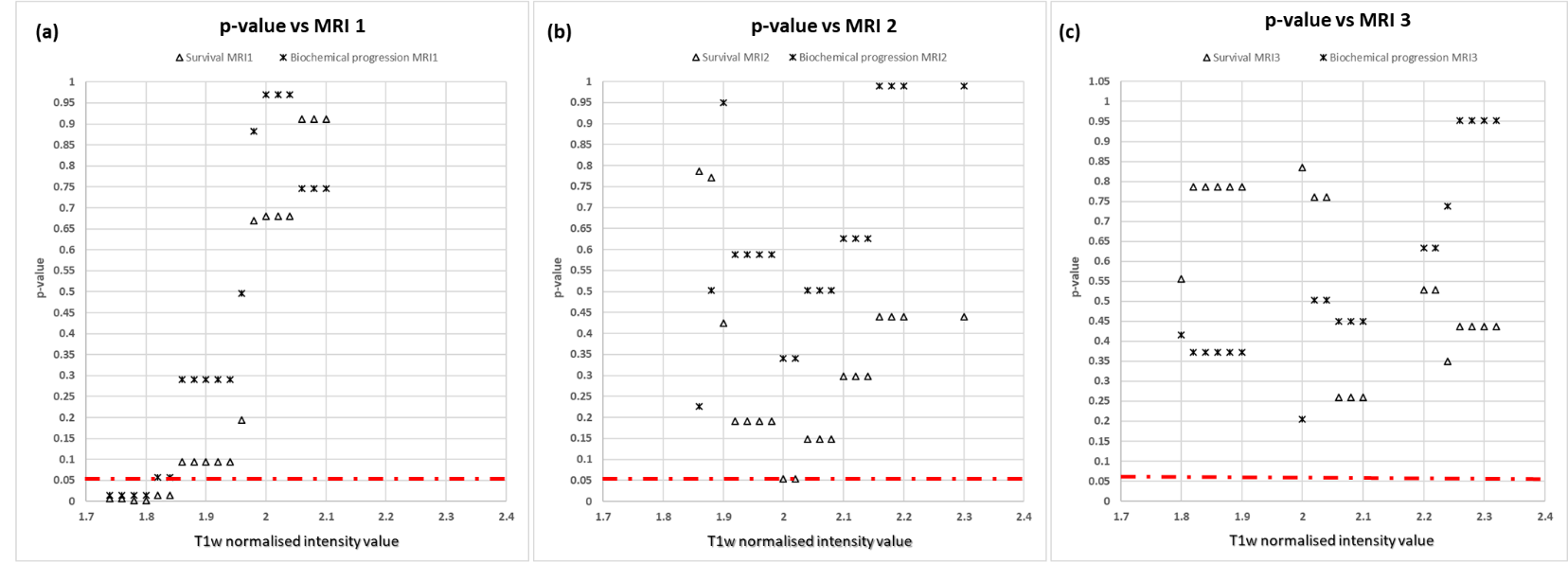


Figure S3: p-value vs MRI intensity splitting value for (a) MRI1, (b) MRI2 and (c) MRI3. Significant splitting values (p-value<0.05) are the ones below the red dash line in the figure.
